# AI-based prediction of pathological risk factors in lung adenocarcinoma from CT imaging: bridging innovation and clinical practice

**DOI:** 10.3389/fonc.2025.1687360

**Published:** 2025-11-13

**Authors:** Yu Huang, Bowen Zhao, Ruiyang Yan, Chi Zhang, Zuhan Geng, Peiyuan Mei, Kuo Li, Yongde Liao

**Affiliations:** 1 Department of Thoracic Surgery, Huazhong University of Science and Technology Tongji Medical College Union Hospital, Wuhan, Hubei, China; 2 School of Computer Science and Engineering, Sun Yat-Sen University, Guangzhou, Guangdong, China

**Keywords:** artificial intelligence, pathology, deep learning, high risk factors, lung adenocarcinoma

## Abstract

Lung adenocarcinoma (LUAD) is one of the main causes of cancer-related mortality worldwide. Pathological risk factors such as spreading through air spaces, high-risk pathological subtypes, occult lymph nodes, and visceral pleural invasion have significant impact on patient prognosis. In recent years, there has been significant progress in the application of artificial intelligence (AI) technology, e.g., deep learning (DL), in medical image analysis and pathological diagnosis of lung cancer, offering novel approaches for predicting the aforementioned pathological risk factors. This article reviews recent advancements in AI-based analysis and prediction of pathological risk factors in lung adenocarcinoma, with a focus on the applications and limitations of DL models, focusing on studies aimed at improving diagnostic accuracy and efficiency for specific high-risk pathological subtypes. Finally, we summarize current challenges and future directions, emphasizing the need to expand dataset diversity and scale, improve model interpretability, and enhance the clinical applicability of AI models. This article aims to provide a reference for future research on the analysis and prediction of pathological risk factors of LUAD and to promote the development and application of AI, especially DL, in this field.

## 1 Introduction

**Figure 1:**
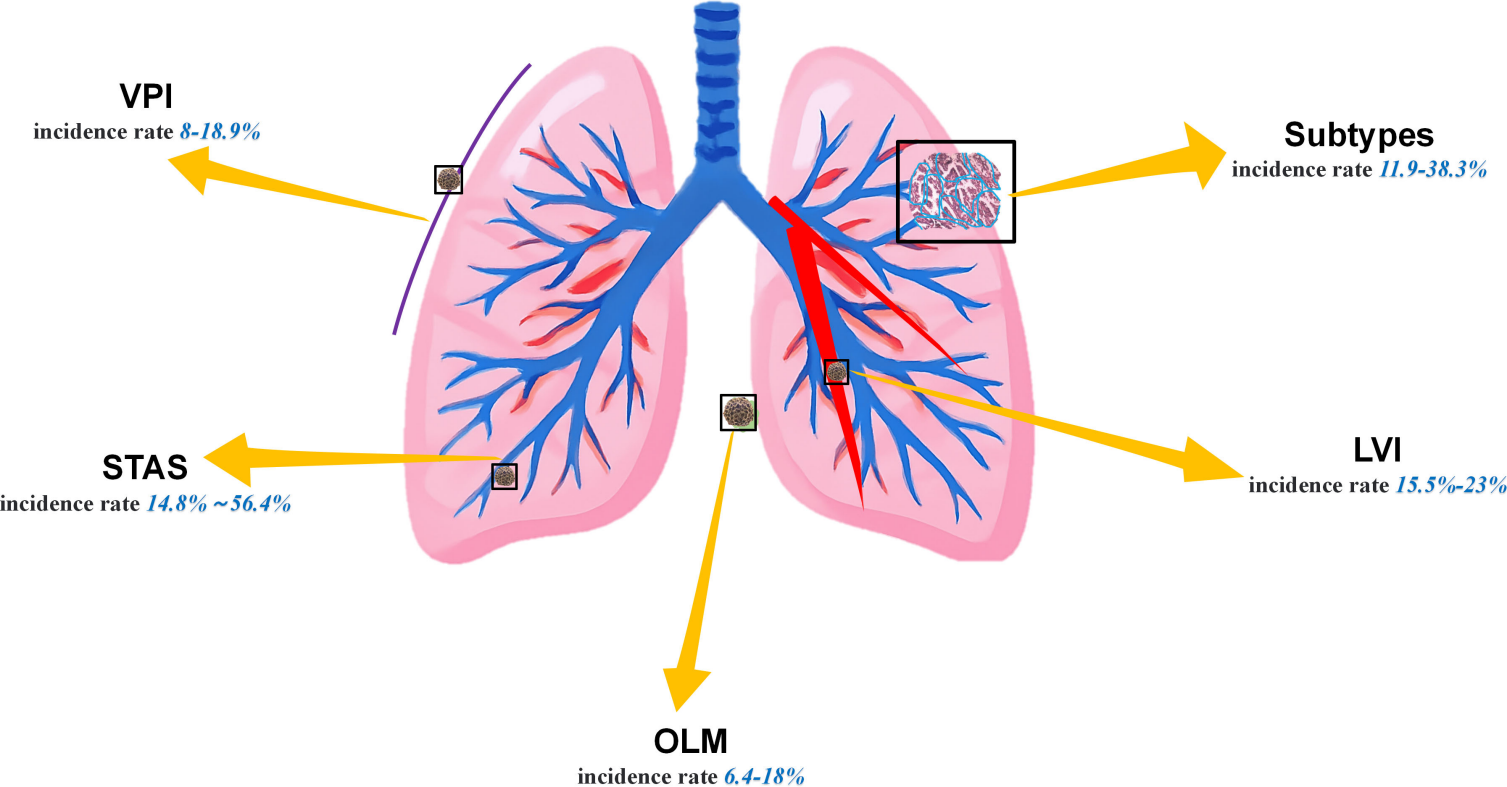
Incidence rates of five high-risk factors (3, 4, 6, 8–11, 13).

Lung cancer remains the leading cause of cancer-related mortality worldwide. Lung adenocarcinoma (LUAD), accounting for over 50% of newly diagnosed lung cancer cases (1, 2), is particularly prevalent. Although surgical resection, as the standard treatment, has significantly improved the prognosis of patients with early-stage LUAD, specific pathological risk factors still contribute to recurrence in certain patients. The currently widely recognized LUAD pathological risk factors primarily include spreading through air spaces (STAS), visceral pleural invasion (VPI), lymphovascular invasion (LVI), occult lymph node metastasis (OLM), and presence of micropapillary and solid subtypes (**Figure 1**) (3, 4). Owing to the presence of these high-risk factors, some subtypes of adenocarcinoma exhibit greater aggressiveness. Although the specific mechanisms remain unclear, the factors may be associated with higher tumor mutation burdens or the presence of microscopic tumor residues (5, 6). Simultaneously, the disruption of intercellular adhesion complexes in the lungs exacerbates the invasive spread of tumors (7). More critically, STAS and subtypes such as micropapillary or solid subtypes often coexist. Patients with a larger number of concomitant high-risk factors are more prone to recurrence and exhibit poorer long-term survival rates (4, 8–10). For such patients, lobectomy with radical lymph node dissection is recommended over segmentectomy (11). Unfortunately, it is not possible to diagnose these factors by visually examining computed tomography (CT) images (12), and invasive preoperative procedures such as bronchoscopy or percutaneous transthoracic needle biopsy (PTNB) may cause harm to patients and offer low sensitivity. Therefore, how to effectively enhance the accuracy of screening for LUAD risk factors through noninvasive methods before surgery, reduce the rate of missed diagnoses, and alleviate the workload of clinicians have become an urgent issue requiring resolution nowadays.

**Figure 2:**
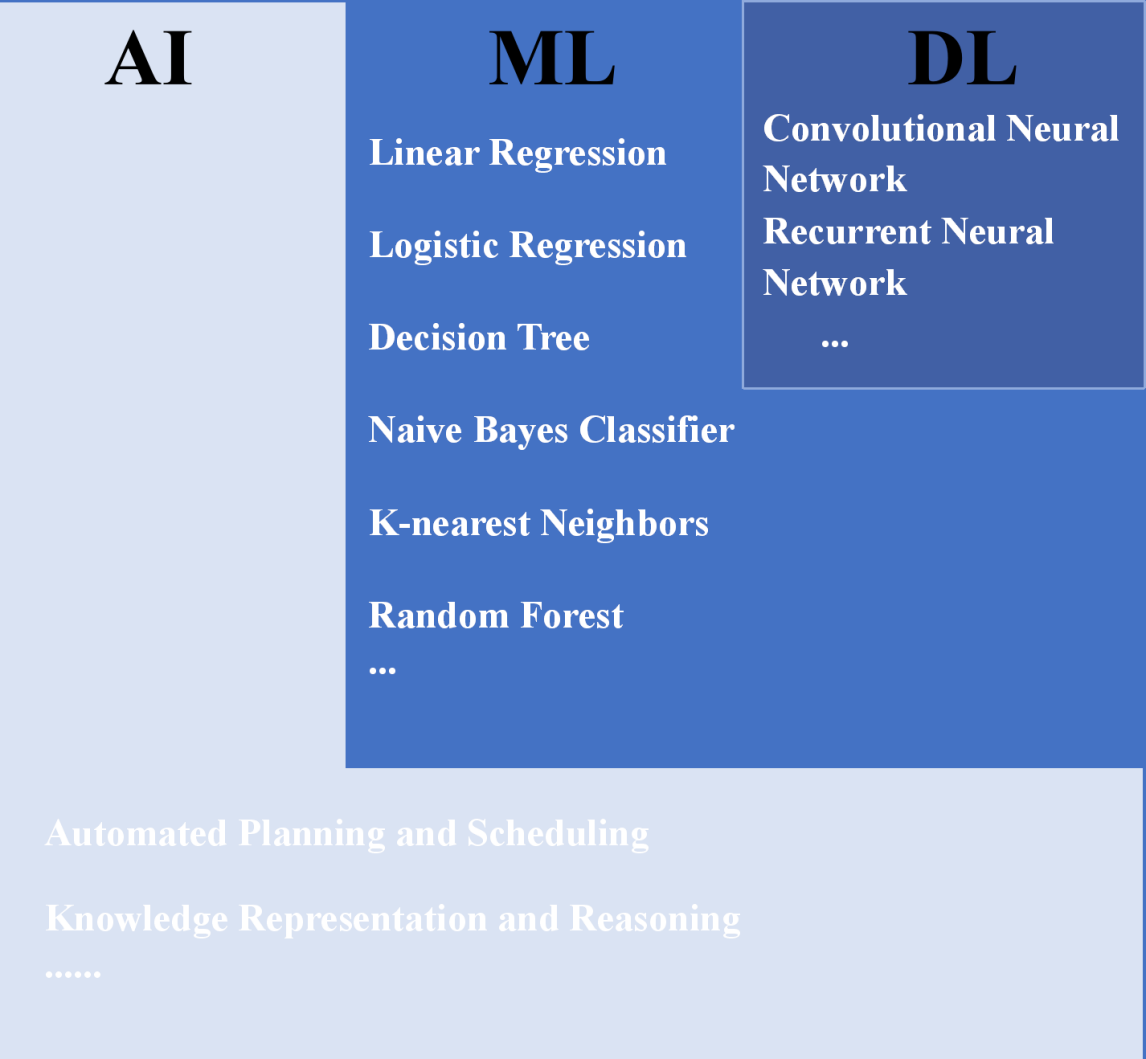
Relationship between AI, ML, DL, and some of their algorithms (14, 24). AI, artificial intelligence; ML, machine learning; DL, deep learning.

Owing to recent advancements in computer technology and statistics, intelligent clinical diagnosis and treatment models for lung cancer, based on tumor imaging, have begun to emerge (13–17). Machine learning (ML) is a primary form of artificial intelligence (AI) technology. Instead of explicit programming, computing systems learn from small- and medium-sized datasets to make predictions or decisions; examples include linear regression and decision trees (18). Deep learning (DL) is a specialized subset of ML, which offers enhanced data volume and model complexity, sacrificing interpretability to achieve extraordinary predictive capabilities from massive datasets (19), e.g., convolutional neural networks (CNNs) (19, 20) and visual transformer (ViTs) (21, 22). The CNNs automatically learn complex features from images through multiple convolutions and pooling operations, thereby enhancing the accuracy and efficiency of lung cancer subtype analysis. Specifically, ResNet is a great landmark architecture of CNN that introduced residual blocks to overcome degradation issues, enabling the training of extremely deep networks (**Figure 2**) (23). Furthermore, ViTs have emerged as another powerful deep learning model for leveraging self-attention mechanisms to capture long-range dependencies and global contextual information within images. The current assumption is that the primary objective of research is to compare the diagnostic capabilities of AI with those of human experts. However, it is evident that classical ML and DL, as complementary AI paradigms, have already demonstrated superior performance and immense potential in terms of speed, reproducibility, and accuracy, surpassing human standards. Given the rapid changes and advancements in this field, this review attempts to summarize the status and trends in the application of AI, especially DL, in the diagnosis and prediction of LUAD risk factors and to discuss current challenges and future perspectives.

## 2 Assessment of high-risk factors in preoperative diagnosis

**Figure 3:**
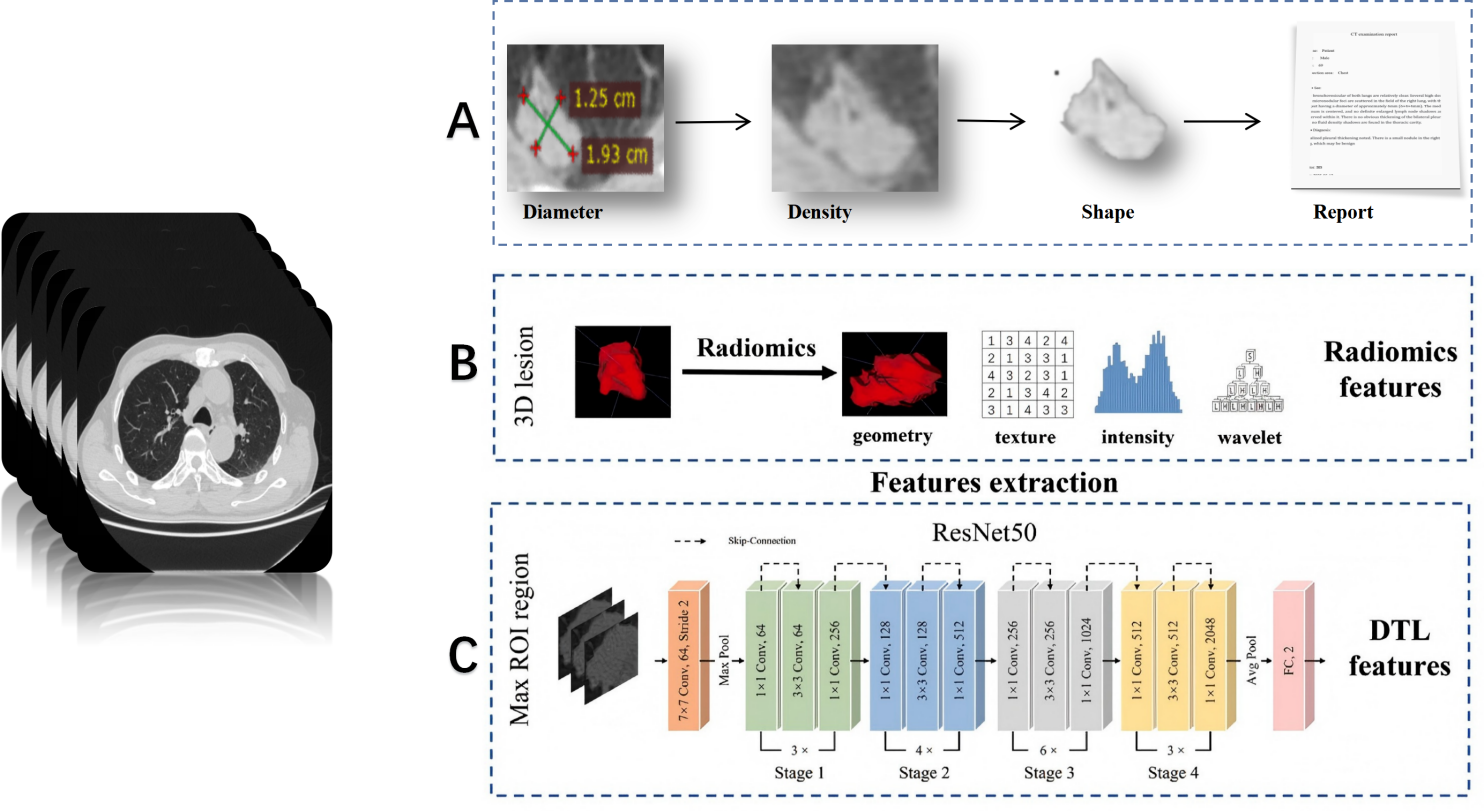
Typical process and differences between clinician **(A)**, radiomics **(B)**, and deep learning **(C)** for lung cancer diagnostic applications (25, 26).

The abovementioned risk factors can only be diagnosed by experienced pathologists based on post-operative slides. However, at this stage, surgical treatment has already concluded, and the surgical approach cannot be altered. Patients with high-risk factors can only receive drug-assisted therapy post-operatively. Therefore, our primary concern is how to enable surgeons to achieve the goals of preoperative visualization of lesions, precise intraoperative resection, and minimized postoperative complications through imaging examinations. Preoperative CT or PET/CT images represent a widely available, easily accessible, and non-invasive source of data. ML is an initial attempt in this area, and DL has become ever more popular in the recent few years (15, 20). **Figure 3** illustrates the fundamental workflow and key components for lung cancer diagnosis of clinician (A), radiomics (B), and deep learning (C).

### 2.1 Spreading through air spaces

STAS is defined as the presence of tumor cells in the air space of the lungs beyond the margins of the existing tumor. It consists of three main forms, namely (1): bronchus filled by microstructures without a central fibrovascular core (2), solid nests in which the air space is filled by solid components of the tumor (3), and bronchus filled by multiple discrete and discrete continuous single tumor cell filling (27). STAS is always indicative of worse prognosis for patients with LUAD (11, 28). Given the significant implications of STAS for lung cancer progression, it has been proposed as a reference for TNM staging (29). Studies said that leveraging AI by interpreting the clinical significance of STAS can enhance diagnostic accuracy or enable risk stratification for STAS-positive patients. This approach will improve clinical workflows and assist physicians in developing appropriate treatment plans (28, 30). Jiang et al. (31) conducted the first study predicting STAS in LUAD using a ML model based on CT imaging. Their results indicated that quantitative parameters (e.g., tumor size, solid composition, and homogeneity) could serve as predictive biomarkers. However, the study’s limited STAS-positive cohort (19.5%) suggests potential data imbalance issues that may compromise the accuracy of the results. Owing to its stronger feature extraction ability and generalization ability, three-dimensional CNN (3D-CNN) offers superior performance to traditional imaging histological models and computer vision models in predicting STAS of non-small cell lung cancer (NSCLC) (32). Features extracted by CNNs from aligned images can be combined with delta-imaging genomics to generate a double delta model (33). Thus, the importance of stereoscopic features and their corresponding delta features in model construction should be confirmed, while extracting a large number of fine features is precisely where DL excels (34). Regarding data sources, unlike the traditional thin-slice CT, PET/CT can simultaneously reflect the morphological and metabolic characteristics of the lesion, thus offering more abundant information (35). Furthermore, maximum standard uptake value (SUVmax) exhibits a higher predictive effect than the maximum tumor diameter in the STAS (36, 37). Therefore, exploration based on DL technology and ^18^F-FDG PET/CT for predicting the existence of STAS is expected (28). Retrospective analyses predicting STAS using AI have become increasingly common, with multiple features such as high-density images confirmed to show a significant association with STAS; yet, how to predict STAS in the patient population with non-solid nodules remains under-explored. We call for more prospective studies on STAS, ideally encompassing a broader cohort of patients with lung cancer.

### 2.2 Lymphovascular invasion

LVI is characterized by the invasion of tumor emboli into the peritumoral lymphatic and/or vascular systems. Consequently, even with complete resection of the primary lesion, the presence of lung cancer cells may still promote recurrence or metastasis via peripheral lymphatics and arteries/veins (8). This explains why LVI is associated with recurrence-free survival (RFS) and overall survival (OS) in NSCLC (8, 38). In 2021, Kyongmin et al. (39) developed and validated a deep cubical nodule transfer learning algorithm (DeepCUBIT) that utilizes transfer learning and 3D-CNN to predict LVI in CT images. In the external validation cohort, DeepCUBIT significantly outperformed single support vector machine (SVM) models that rely solely on tumor size or C/T ratio, demonstrating the informational value of peritumoral regions in CT images. Specifically, in some nodules with C/T ratios below 1.0, the main feature for the prediction comes from the tumor margins—the interface between the tumor and adjacent lung parenchyma. Therefore, we suggest that future studies should consider reasonable expansion in lung nodule image segmentation to incorporate more information. Furthermore, integrating clinical and radiological data can also enhance the accuracy of LVI prediction (40). Compared with simple models, two-dimensional and three-dimensional CT imaging features and clinical radiological data can more accurately reflect the true presence of lung cancer. Of course, the integration of expanded multi-center datasets is equally essential to optimize the model’s learning capabilities (41).

Moreover, the analysis of LVI prediction relevance and interpretability is crucial, as a model that merely reports LVI positivity, without disclosing the reason for the result, cannot deliver satisfactory clinical performance. This necessitates further validation to confirm the efficacy of customized deep network features in capturing imaging phenotypes beyond known histological characteristics (39, 42).

### 2.3 Visceral pleural invasion

VPI can reduce the 5-year survival rate of patients in stage IA by 16% (8, 10). Therefore, VPI not only reflects the severity of tumor invasiveness but is also regarded as an independent prognostic indicator (10). Regrettably, when thoracic surgeons combined clinical information, preoperative CT scans, and intraoperative pleural biopsies, the diagnostic accuracy rate of VPI was found to be no more than 60% (43). In contrast, DL can automatically learn useful feature representations from lung nodes of raw data, significantly improving the efficiency and accuracy of diagnosis (44, 45). Choi et al. developed a VGG-16 model that achieved an area under the curve (AUC) of 0.81, although its accuracy was constrained by the small sample size (*n* = 212) (44). In addition to the issue of sample size, the heterogeneity of diagnosis also affects the stability of the model (46). However, other researchers remain optimistic about the future of this field—for instance, one study found that images based on high-resolution computed tomography (HRCT) could effectively predict VPI (45). Another study developed a 3D-ResNet DL model and found that pleural traction angle >30° and disappearance of subpleural fat space were important predictive indicators. The AUC of this model in the training, internal validation, and external validation queues was around 0.70 (46). Tsuchiya’s team used thoracoscopic images and AI to identify the presence of VPI in clinical stage I LUAD and found that the AI model based on ViTs achieved a sensitivity of 0.85 in real-time VPI recognition (47). This study therefore suggests another approach to acquiring the ability to intraoperatively diagnose high-risk factors from images taken by intraoperative thoracoscopy as well, rather than relying solely on preoperative CT. The VPI prediction model holds great promise and significant clinical value, as relying solely on the distance between nodules and the pleura in CT images to diagnose VPI carries risks; in other words, the presence of pleural–tumor contact does not always indicate VPI. Conversely, tumors can invade the pleura through retraction even without direct contact (48). Therefore, a stronger collaboration between radiologists, surgeons, and pathologists is recommended to foster multidisciplinary consensus and address challenges in this field.

### 2.4 Occult lymph node metastasis

OLM refers to the situation where preoperative CT is not showing lymph node enlargement, but postoperative pathological results do confirm the existence of tumor metastasis (49). However, the fact that radiologists may not detect lymph node abnormalities based on the images does not necessarily indicate that no abnormal manifestations exist on the images—in many cases, these abnormalities are too small to be observed by eye. Therefore, at present, experienced radiologists spend a considerable amount of time carefully examining tumor morphology and lymph node manifestations before diagnosing few OLM cases (50). In addition, by integrating parameters such as carcinoma embryonic antigen (CEA) (>5 ng/mL) and the proportion of nodular solid components (consolidation-to-tumor ratio, CTR ≥0.7), 83% of N1 metastatic cases can be identified (51). AI models based on architectures such as XGB have demonstrated excellent performance in predicting OLM in patients with clinical stage IA LUAD (52–54). The pulmonary nodule volume-doubling time (VDT) of patients with LUAD is shorter when solid/microform components are dominant, which leads to a higher metastasis rate; therefore, VDT or mass doubling time (MDT) may be effective predictors of OLM among NSCLC (55, 56). In other words, when establishing a DL model, considering CT follow-up results for more than 1 year is conducive to a better model (55).

PET/CT has high sensitivity in detecting hilar and mediastinal lymph nodes. Cytokeratin fragment (CYFRA) 21-1 (>2.46) and liver SUVmax (L-SUR) (>2.68) have been proven to be independent risk factors for OLM (57). Furthermore, the use of PET/CT data to build AI models is feasible in predicting OLM and offers high sensitivity and specificity in prospective tests (58). PET/CT-DL models have achieved excellent performance, respectively, in the prediction of latent N1 and N2. High-risk patients identified by this model can benefit from lymph node biopsy, lobectomy, and adjuvant therapy (59). It is important to note that although the images are necessary for analysis, not all PET/CT devices have the ability to output such high-quality images. Furthermore, PET/CT is not commonly used for the diagnosis of ground-glass lung nodules ≤2 cm (35, 60) and is often not the preferred test in developing countries, such as China, owing to its high cost. This may reduce the clinical applicability of such models in some institutions. Because of the low incidence of occult lymph nodes (approximately 5%–15%) (6), the imbalance in samples caused by a low number of positive cases limits AI’s learning capacity. Furthermore, while the study targets metastatic lymph nodes, preoperative CT scans struggle to accurately diagnose and delineate them. Consequently, recent AI predictions still focus on pulmonary nodules as regions of interest—a contradiction that appears difficult to resolve. Additionally, an increasing number of studies are shifting their focus to N1 and N2 metastases rather than simple binary classification problems, which better align with actual clinical needs regarding lung cancer diagnosis and treatment.

### 2.5 High-grade pathological subtype (micropapillary or solid pattern)

During the development of LUAD, various growth patterns (including squamous, acinar, micropapillary, and solid types) occur. Generally, various pathological subtypes are calculated in increments of 5%, and the proportion of the main subtypes determines the dominance of the pathological types (3). We focused on the micropapillary subtypes and solid types in LUAD, which are closely related to DFS and OS after surgery (61) and accompanied by a higher postoperative recurrence rate (9, 62). More researchers are exploring the application of DL techniques in predicting LUAD pathotypes due to DL detection of details that cannot be observed by the human eye (63). Dong et al. developed two deep learning models based on LeNet and DenseNet, discovering that deep learning techniques can not only be applied to the classification of invasive LUAD but also used to predict micropapillary patterns—marking the first attempt at utilizing deep learning technology in this field (64). Chang et al. used an advanced LUAD subtype detection algorithm to analyze near-pure lung adenocarcinoma imaging with histological features and plaque images and obtained high sensitivity and moderate specificity (65). As intratumoral heterogeneity is manifested in the pathological components and tumor imaging features, a single modality may not be able to accurately identify the solid components within the tumor (66–68). Thus, the establishment of a comprehensive model combining DL-based scoring and clinical imaging features to distinguish LUAD with micropapillary and solid-type structure enhanced the classification performance (68). Chen et al. developed a DL model based on the data of 502 patients with pathologically diagnosed high-grade adenocarcinoma within 4 years. They applied a solid-attenuation-component-like subregion masks (tumor area ≥-190 HU) to guide the DL model in analyzing high-grade subtypes. This is another promising preoperative prediction method for high-grade adenocarcinoma subtypes (69). Similar to the assessment of other high-risk factors, ^18^F-FDG PET/CT data are increasingly being applied to DL because of the high metabolic rate of tumor cells and have demonstrated decent predictive accuracy for microscopic or solid components within LUAD (70, 71). A new study demonstrated the combined DL of texture features for ultra-short echo time MRI (UTE-MRI) to avoid radiation exposure while maintaining comparable diagnostic performance with CT for subtypes such as micropapillary (AUC = 0.82 vs. 0.79) (72). Despite the small sample size of the study (74 lesions), this implies the feasibility of the UTE-MRI DL model with the significant advantage of UTE-MRI in avoiding radiation hazards, especially for patients requiring multiple follow-up visits over time and for children with radiation sensitivity. Furthermore, various imaging examinations and large models that integrate imaging and genomics are providing new perspectives and tools for personalized treatment and surgical decisions. Both clinical and quantitative radiomics features, along with DL-based scoring models, can decode LUAD phenotypes. However, a notable issue arises: LUAD with multiple subtypes may exhibit mixed subtype characteristics rather than distinct information from the three subtypes, potentially limiting the discriminative capability of AI processing. Most studies rely on quantitative analysis of specific subtypes using thresholds such as 5%, 10%, or 20%. Future DL models and related fusion models require a more detailed stratified analysis beyond qualitative predictions (**Table1**).

**Table 1:** Application of AI in the prediction of pathological risk factors.

High-risk factors	Study	Total cases	Positive cases	Algorithm	AUC^a^
Spread through air spaces	Jiang et al. (31)	462	90	Random forest	0.754
	Tao et al. (32)	203	89	3D-CNN	0.800
	Jin et al. (33)	585	143	Delta-DL	0.840
	Lin et al. (34)	581	89	DL	0.820
	Gao et al. (36)	466	113	Logistic regression	0.786
Lymphovascular invasion	Beck et al. (39)	695	254	3D-CNN	0.717
	Liu et al. (40)	2077	299	3D-Resnet-9	0.770
	Wang et al. (41)	3034	106	Dual-Head Res2Net_3D23F	0.869
Visceral pleural invasion	Choi et al. (44)	817	256	3D-CNN	0.750
	Kudo et al. (45)	472	224	EfficientNet V2−M	0.780
	Lin et al. (46)	2077	381	3D-ResNet-9	0.690
	Shimada et al. (47)	127	49	Vision Transformer	0.840
Occult lymph node metastasis	Liu et al. (52)	258	129	XGB	0.917
	Tian et al. (53)	1325	478	ResNet-18	0.754
	Karita et al. (55)	560	89	Logistic regression	0.768
	Liu et al. (56)	144	27	Logistic regression	0.860
	Zhong et al. (59)	3265	655	ResNet-18	0.914
Micropapillary and solid subtypes	Chen et al. (65)	158	42	Logistic regression	0.86 ± 0.01
	Wang et al. (66)	111	31	CNN	0.861
	Xing et al. (67)	273	61	Logistic regression	0.843
	Wang et al. (68)	512	191	Wide residual network	0.827
	Chen et al. (69)	502	180	SACA-DL	0.930

a Performance evaluation in test data or external validation data.

3D-CNN, three-dimensional convolutional neural network; DL, deep learning; XGB, extreme gradient boosting; SACA-DL, solid attenuation components attention deep learning model.

## 3 Evaluation of high-risk factors combining pathological tissue and CT

Multimodal fusion has become an inevitable direction for AI-assisted lung cancer diagnosis. Pathological images serve as another critical information source alongside radiological imaging—for example, the ResNet model trained on whole-slide images demonstrates recognition capabilities comparable to those of senior pathologists in identifying pathological subtypes of LUAD (73). In recent years, the integration of CT and pathological information is more commonly used to predict patient prognosis. However, Wu and colleagues constructed a predictive model using preoperative CT images and intraoperative frozen section results to assess the extent of lung cancer invasion (74). This represents a novel clinical issue: although diagnosis based on intraoperative frozen section offers high specificity to identify high-risk factors in LUAD, its sensitivity remains limited (75). DL models analyze feature maps and perform logical reasoning, exhibiting decision-making processes consistent with the diagnostic reasoning of pathologists. They even demonstrate superior capabilities in identifying minute lesions (76). This demonstrates that AI models can now automatically detect and quantitatively analyze STAS, acinar, micropapillary, papillary, solid subtypes, and normal tissue in final histopathological sections, achieving satisfactory results (77). Thus, leveraging AI to further enhance the diagnostic accuracy of frozen section analysis has a solid practical foundation. Going a step further, the question of whether integrating preoperative CT imaging with preoperative needle biopsy specimens, bronchoscopic biopsy specimens, or intraoperative frozen section results to construct a more comprehensive and integrated intelligent model could achieve more precise predictions warrants in-depth exploration.

## 4 The future of prediction of high-risk factors for LUAD based on DL

**Figure 4:**
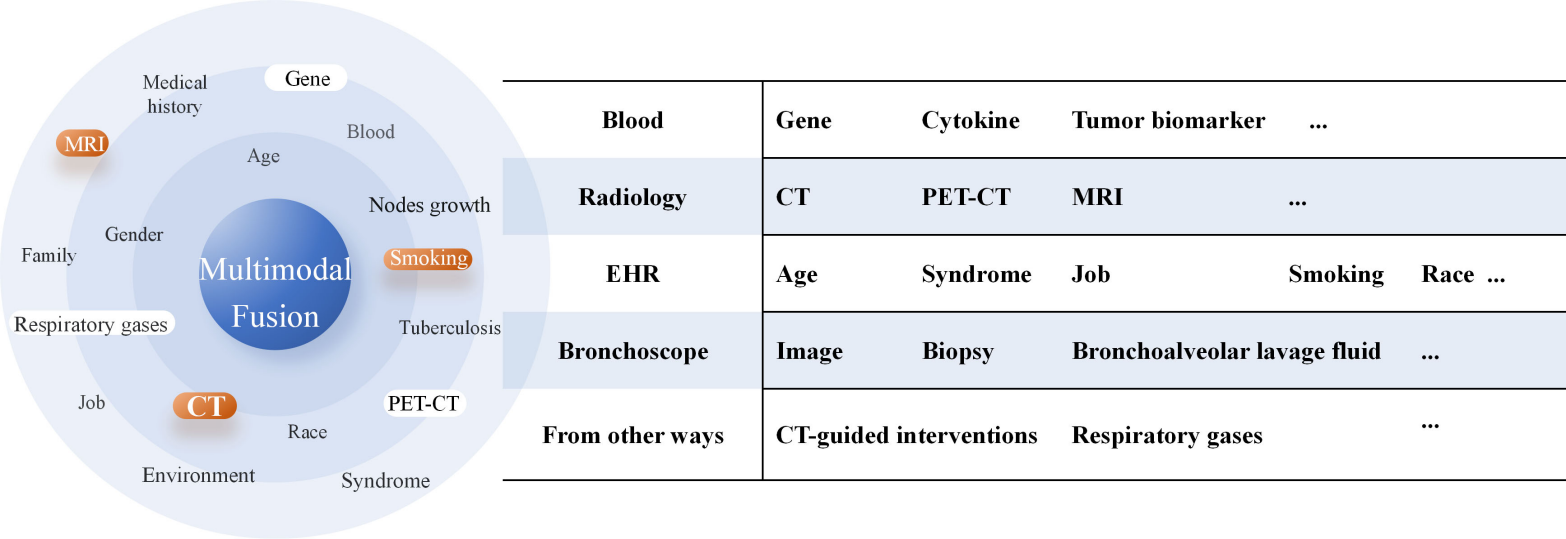
Multimodal data types and sources available for artificial intelligence models.

DL offers advantages such as high efficiency, accuracy, and automation, making its potential self-evident. However, several key challenges remain, including technical issues such as sparse datasets and model generalization. In medical applications, AI models must also demonstrate high interpretability to gain the trust and acceptance of clinicians. Therefore, we believe that future research directions may include the following:

1. In predicting high-risk factors for LUAD, the core value of multimodal fusion models lies in their ability to integrate heterogeneous medical data, break down information barriers, and ultimately enable more precise and personalized prognostic assessments. By integrating imaging data such as CT, PET/CT, and pathological images from the puncture surgery or bronchoscopy and electronic health records (EHRs)—including the age, symptoms, and smoking history (**Figure 4**)— multimodal models can construct multidimensional disease views, providing a more comprehensive reflection of lung cancer progression stages and individual patient condition. The adoption of large language models further facilitates faster and more standardized generation of EHRs in clinical practice, enhancing the input quality for multimodal systems (78), and significant progress has been made in validating the feasibility and potential of multimodal strategies—for instance, She et al. (59) developed a multimodal DL model based on PET/CT that demonstrated a significantly higher accuracy in diagnosing OLM relative to single-modality DL models, clinical models, and physicians. Chen et al. (79) established a multiomics model (clinic-RadmC) integrating clinical and radiomic data with circulating cell-free DNA fragmentomic features in 5-methylcytosine (5mC)-enriched regions to predict the malignancy risk of indeterminate pulmonary nodules. Moreover, a combined DL model incorporating clinical, imaging, and cell-free DNA methylation biomarkers has been shown to accurately classify pulmonary nodules, potentially reducing approximately 80% of unnecessary surgeries and delayed treatments (80).However, the construction and application of multimodal models still face several critical challenges. First, significant differences in format, dimensionality, and semantic levels exist between different modal data, making effective modal alignment and deep fusion a persistent technical hurdle. Second, the inherent “black-box” nature of DL models makes it difficult for clinicians to understand which features—such as image regions or gene mutations—underlie the model’s high-risk assessments.

2. In the field of medical imaging, some phenomena, such as occult lymph nodes whose incidence rate is approximately 10%, are not common. Moreover, the manual annotation of CT and pathological images is costly and time-consuming, and the limited availability of annotated datasets makes small-sample learning a key challenge. To address this issue, researchers have explored various strategies. Data augmentation techniques, including geometric transformations, intensity perturbations, and advanced methods such as MixUp and CutMix, enrich the diversity of training and reduce overfitting (81, 82). Few-shot and meta-learning paradigms offer another promising direction, wherein models are trained in a batch manner to learn transferable representations, enabling rapid adaptation to new cohorts or rare subtypes with only a few examples (83, 84). Knowledge distillation from large teacher models to lightweight student models has also been used to mitigate overfitting and improve sample efficiency (85). Overall, these methods aim to overcome the inherent limitations of small-sample medical datasets and support more robust and generalizable DL models.

Furthermore, the latest advancements in large-scale pre-trained models show great promise. Models such as CLIP, SAM, and DINOv2, which were initially developed for natural images, have demonstrated significant potential in the field of medical imaging, particularly in few-shot adaptation, interactive annotation, and cross-modal integration (24, 86, 87). These developments collectively suggest that customizing model designs for the characteristics of medical data and transferring from foundational models will also play a crucial role in overcoming the inherent limitations of medical datasets.

3. The application of AI in clinical practice relies heavily on evidence-based medical support obtained through rigorous clinical trials to validate its efficacy, safety, and reliability. Most previously mentioned studies are predominantly retrospective analyses, although some high-quality studies have included prospective validation cohorts. Most published studies have focused on the East Asian populations (such as patients from China, Japan, and South Korea), thereby presenting geographical and demographic limitations. To address the issue of generalization of the capabilities of AI models across diverse populations, regulatory bodies such as the US Food and Drug Administration (FDA) and the European Medicines Agency (EMA) are actively promoting multicenter trials and external validation mechanisms to assess their actual efficacy in heterogeneous populations (88, 89). However, it is encouraging that the clinical research findings on AI published to date have been almost entirely positive. A systematic review of 39 randomized controlled trials (RCTs) conducted as of July 2021 revealed that 77% (30/39) of AI interventions demonstrated superior efficacy relative to standard clinical care and 70% (21/30) showed clinically meaningful improvements in outcomes, with radiology studies constituting the majority of these findings (90). Other prospective studies have also demonstrated that CT-based AI tools can enhance the sensitivity of lung nodule diagnosis (90–92). However, no further studies with higher levels of evidence have been published on the prediction of high-risk factors for LUAD. Despite its promise, AI still faces multiple challenges in clinical applications, including data privacy, system security, and interoperability. Future research should include multicenter, large-scale, rigorously designed clinical trials to fully validate the effectiveness of AI—particularly DL models—in real-world clinical settings. Simultaneously, collaboration between clinicians, AI companies, and regulatory bodies must be enhanced to jointly address complex ethical and legal issues, thereby promoting the robust and responsible integration of AI within the healthcare sector.

## 5 Conclusions

AI-based research on the prediction of high-risk factors for LUAD shows great potential in medical image analysis and pathological diagnosis. DL models significantly improve the accuracy and efficiency of early prediction and diagnosis of lung cancer by automatically identifying and classifying high-risk factors in CT images. However, current techniques still face challenges such as insufficient datasets, high model complexity, poor interpretability, and data privacy. Future research should focus on translating cutting-edge technologies into clinical practice through standardized data collection, lightweight model architectures, and rigorous evidence-based validation. With the introduction of new architectures such as transformers, AI is expected to become an important method to support decision-making in the diagnosis of high-risk factors and treatment of LUAD.
